# Learning neural connectivity from firing activity: efficient algorithms with provable guarantees on topology

**DOI:** 10.1007/s10827-018-0678-8

**Published:** 2018-02-20

**Authors:** Amin Karbasi, Amir Hesam Salavati, Martin Vetterli

**Affiliations:** 1 Inference, Information and Decision Systems Group, Yale Institute for Network Science, Yale University, New Haven, CT 06520 USA; 2 Laboratory of Audiovisual Communications (LCAV), School of Computer and Communication Sciences, Ecole Polytechnique Federale de Lausanne (EPFL), Lausanne, Switzerland

**Keywords:** Functional connectivity, Synaptic connectivity, Neural signal processing, Neural network

## Abstract

The connectivity of a neuronal network has a major effect on its functionality and role. It is generally believed that the complex network structure of the brain provides a physiological basis for information processing. Therefore, identifying the network’s topology has received a lot of attentions in neuroscience and has been the center of many research initiatives such as Human Connectome Project. Nevertheless, *direct* and invasive approaches that slice and observe the neural tissue have proven to be time consuming, complex and costly. As a result, the *inverse* methods that utilize firing activity of neurons in order to identify the (functional) connections have gained momentum recently, especially in light of rapid advances in recording technologies; It will soon be possible to simultaneously monitor the activities of tens of thousands of neurons in real time. While there are a number of excellent approaches that aim to identify the functional connections from firing activities, the scalability of the proposed techniques plays a major challenge in applying them on large-scale datasets of recorded firing activities. In exceptional cases where scalability has not been an issue, the theoretical performance guarantees are usually limited to a specific family of neurons or the type of firing activities. In this paper, we formulate the neural network reconstruction as an instance of a graph learning problem, where we observe the behavior of nodes/neurons (i.e., firing activities) and aim to find the links/connections. We develop a scalable learning mechanism and derive the conditions under which the estimated graph for a network of Leaky Integrate and Fire (LIf) neurons matches the true underlying synaptic connections. We then validate the performance of the algorithm using artificially generated data (for benchmarking) and real data recorded from multiple hippocampal areas in rats.

## Introduction

Reconstructing the connectivity of neuronal networks has been a major challenge for the past decade. Currently, the only reliable way to map the underlying *synaptic connectivity* of neuronal networks is by using invasive procedures, which are prohibitively complex and time-consuming: it took more than 10 expert-year to map the whole connectome of *C. Elegans*, comprising only 302 neurons and 7283 synaptic connections (Watts and Strogatz 1998). Similarly, a 10 expert-year effort was required to capture the connectome of *fruit fly* medulla columns, with only 379 traced neurons and 8637 synapses (Plaza et al. 2014). To map the whole brain of a fruit fly, with around 10,000 neurons, we would have to spend around 4700 expert-year (Plaza et al. 2014; Chiang et al. 2011). Following the same approach and using the current tech- nology, it is estimated that it will take around 14 billion man/year to completely map the human brain’s connectome (Plaza et al. 2014). Although there is an increasing effort to make some parts of the invasive procedures automated, such approaches remain impractical even for mid-sized networks. Furthermore, the current invasive techniques cannot be applied to live specimen.

In contrast, *inverse* methods with the focus on mapping the *functional* connectivity from the activity of the neurons have received more attention in recent years. These approaches are non-invasive (or minimally invasive) so they can be applied to live specimen and they require much less time and labor to identify the functional network. Furthermore, rapid advances in recording technologies has made it possible to simultaneously monitor the activities of tens (Perin et al. 2011) to hundreds of neurons (Buzsáki 2004; Grewe et al. 2010). Upcoming technologies will significantly improve the accuracy and scale of recording neurons’ activities. It is worth mentioning that there has also been significant progress in simultaneously recording and stimulating a set of neurons (Khodagholy et al. 2014; Herrera and Adamantidis 2015; Bertotti et al. 2014). These advancements provide an abundance of data for which computationally efficient and accurate inverse algorithms would be welcome.

In this paper, we focus on the inverse problem. Our main goal is to design efficient and *scalable* algorithms that result in good approximations of the underlying *synaptic graph*. In other words, we are interested in algorithms whose inferred functional network is a close match to that of the underlying synaptic connectivities for a group of Leaky Integrate-and-Fire (LIf) neurons (Gerstner and Kistler 2002).

To this end, we apply a technique, usually known as the *kernel method* in the machine learning literature, to map the nonlinear inference problem to a linear equivalent in the kernel space. Then, we formulate the network inference problem as an instance of a constrained optimization problem where the objective function has a simple form and the constraints are all linear. As a result, we develop an algorithm that easily scales to large datasets of recorded neural activities. Moreover, we mathematically analyze this mapping and derive the conditions under which our proposed algorithm successfully identifies the *type* of underlying synaptic connections (e.g. being excitatory/inhibitory) in the limit of large available data.

We also show that the proposed technique is equally applicable to networks of both deterministic or stochastic neurons that follow the widely used LIf model. We support our theoretical findings with an exhaustive set of simulations where we validate the performance of our algorithm with respect to the ground truth networks (in artificially generated spiking data where the ground truth is available). We also report the result of our algorithm applied to a datatset of firing activities recorded from hippocampal areas in rats (Mizuseki et al. 2013). We find that our results are quite in line with previous findings (Mizuseki et al. 2009).

**Fig. 1:**
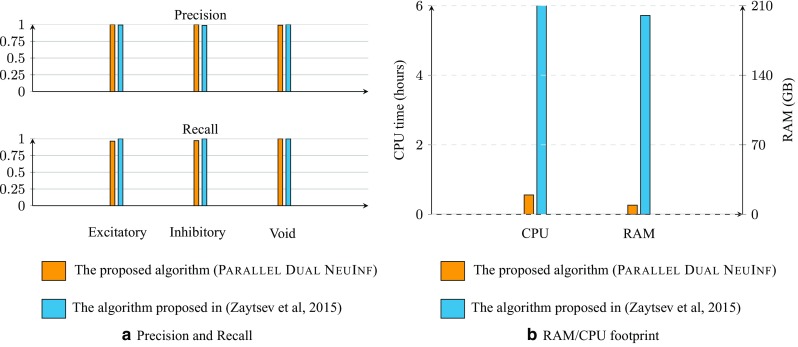
Performance of our proposed algorithm in identifying the *type* of neural connections in a network of 1000 LIf neurons for a dataset of artificially generated spiking activities (courtesy of Zaytsev et al. (2015)) **(a)** and the memory/CPU footprint of our proposed algorithm compared to a similar approach proposed in Zaytsev et al. (2015) over the same dataset

Figure 1 summarizes the main *computational framework* of this paper: in Fig. 1a we show that in the limit of large data, the proposed algorithm can successfully identify the type of synaptic connections, whereas Fig. 1b demonstrates the memory/CPU footprint of the proposed approach compared to that of the approach based on Generalized Linear Models (GLM) (Zaytsev et al. 2015).^1^

## Related work

Solving inverse problems and trying to reverse engineer neural circuits have long been one of the main research topics in neuroscience. On a single neuron scale, characterizing neurons response and predicting its output spikes based on the input stimuli has been one of the highly explored issues and methods based on white noise analysis have been used extensively with remarkable results (see Pillow and Simoncelli (2003) for a recent example). Methods based on integrate-and-fire model for neurons have also been extensively used to infer mathematical models of neural circuits using the pre and post-synaptic data. Lazar and Slutskiy (2014) is a nice example of such approaches, where the Hodgkin-Huxley model is used to identify the neural circuit. Nevertheless, this approach requires that the pre and post synaptic measurements of the target neuron be available.

Moving to identifying the network connectivity, Cross Correlogram is perhaps the most widely-used method to identify (functional) connection between pairs of neurons or regions (Brown et al. 2004). However, approaches based on Cross Correlogram usually fall short of identifying *causal* relation or *effective* connectivity of neurons. It is very well established in statistics that the existence of correlation between two events is neither a necessary nor a sufficient conditions for inferring causality. This is why statistical hypothesis tests such as *Granger causality measure* were proposed as an alternative in order to overcome the drawbacks of Cross Correlogram (e.g., Kim et al. (2011)).

Another recent line of work has primarily focused on inference methods that are tailored to LIf model of neurons. In particular, Van Bussel et al. (2011) convert the non-linear firing behavior of LIf neurons into a set of linear equations, which can be solved given a sufficient number of recorded samples. While being efficient, this algorithm is highly sensitive to the accuracy of spike times and relies on the knowledge of model parameters (e.g. synaptic propagation delays) which are difficult to obtain. Memmesheimer et al. (2014) and Baldassi et al. (2007) proposed an inference algorithm based on the Perceptron learning rule. Furthermore, Memmesheimer et al. (2014) proved that under accurate estimate of spike times it is possible to identify a simple *n*-to-1 feed forward network. They also proposed a heuristic extension that works with *finite precision* in recorded spike times. Nevertheless, their model does not take the (random) synaptic delays into account. Moreover, having extra post-synaptic neurons even in a simple feed forward scenario can have a dramatic effect on the performance of the inference algorithm when the structure of the graph (i.e., here being feed-forward) is not known a priori. In Monasson and Cocco (2011) two Bayesian approaches are proposed to find the connections in a network of LIf neurons. Nevertheless, the proposed approaches do not account for the (random) synaptic delays as well as the effect of hidden neurons. Furthermore, the algorithm highly depends on accuracy of the recorded spike times.

A more complex and accurate family of approaches rely on Generalized Linear Models (GLM) (Paninski 2004). These methods consider the collective activity of the neural group and focus on finding the best functional network that can explain the activity. GLM was recently used in reconstructing a real physiological circuit from recorded neural data (Gerhard et al. 2013) as well as reconstructing the functional connectivity for the ganglion cells in the retina (Pillow et al. 2008). The approaches that are based on GLM are generally accurate (i.e. they identify the correct set of connections in the underlying graph) *provided* that the neural model used to generate the spike data matches exactly the one used in GLM (Ramirez and Paninski 2014). Extending these methods to exploit the prior distribution on the neural connections results in effective Bayesian models that are especially powerful in the face of limited data. In particular, Stevenson et al. (2009) proposed a Maximum a Posteriori (MAP) estimate to infer the neural connections and reported highly accurate results in limited data regime at the expense of very high computational costs. Bayesian approaches have also been used in identifying connections directly from calcium-imaging data (Mishchenko et al. 2011).

In light of the aforementioned advantages, GLM-based techniques are among the favorite state-of-the art approaches. Nevertheless, they are not without limits. The first and probably most important drawback is scalability, which makes handling large datasets, both in terms of number of neurons and duration of recorded firing activity, difficult. Recently, however, several approximations have been suggested to resolve this issue (Ramirez and Paninski 2014; Zaytsev et al. 2015). Nevertheless, these approximations work only for a particular choice of nonlinearity (Zaytsev et al. 2015) and similar to GLM-based techniques, the convergence is only guaranteed when the model for neurons and that of GLM’s closely match each other. Soudry et al. (2015) have proposed an approach that covers a wider set of nonlinearties to overcome this issue to some extent. However, random synaptic delays have not been addressed and no guarantees are provided on the performance of the proposed method.

Close to GLM-based methods are recent approaches that model the stochastic firing rates by a Hawkes point process. In contrast to a (homogenous) Poisson process, which assumes that events occur independently of one another, in a Hawkes process past events can increase (e.g., excitation of neurons) or decrease (e.g., inhibition of neurons) the probability of future events. Based on this parametric assumption, Moore and Davenport (2015) identified the connections in a medium-sized network while assuming that the traffic is generated according to a Hawkes process. Similarly, Hall and Willett (2016) aimed at inferring the connections as well as predicting the firing rate of neurons based on their past firing activity through an online learning algorithm. Nevertheless, both methods heavily rely on the assumption that the traffic is generated according to a Hawkes process, whereas we make no such statistical assumptions. Moreover, neither of the approaches take the effect of hidden neurons into account or evaluate the performance of their algorithms in scenarios where inhibitory connections are present.

In this paper, we propose a novel learning approach in identifying the functional connections which offers the following properties:

1. **Scalability**: from a practical point of view, it allows better scalability (in contrast to previous work), i.e., it requires less memory and can scale with limited resources available (see Fig. 1).
2. **Performance guarantees**: from a theoretical point of view, its performance guarantees hold under a larger family of neurons and nonlinearities.
3. **Hidden neurons**: the simplicity of the approach also enables us to derive the sufficient conditions under which the estimated functional network returned by our algorithm is not affected by the existence of hidden neurons and matches the underlying synaptic graph in the limit of large data.

Finally, we should mention that the *consistency problem* even for a *n*-to-1 feed forward network is NP-hard. In words, determining whether or not there exists a set of delays and weights such that we can fully match the set of input firing patterns to the output is very difficult (Maass and Schmitt 1999) . Although this result does not necessarily imply that finding such a configuration is impossible (under the right set of conditions), it shows that finding provable ”positive learning results” for the case of spiking neurons is quite challenging.

## Model formulation and problem statement

We formally introduce the neural models and the network structures considered throughout the paper. We also formally state the network inference problem.

### **Neurons’ model**

We first consider a network of *deterministic* but *noisy* Leaky Integrate and Fire (LIf) neurons with a *fixed* firing threshold *𝜃* (Gerstner and Kistler 2002) . In this model, the membrane potential of a given neuron at time *t* is described by

1 $$ h(t) = h_0 + \sum\limits_{i = 1}^n g_i K_i(t) + v(t), $$

where *h*_0_ is the baseline voltage, *g*_*i*_ is the actual synaptic weight (i.e. the ground truth) of the incoming connection from the pre-synaptic neuron *i*, *K*_*i*_(*t*) is the accumulated effect of neuron *i* on the post-synaptic neuron at time *t*, and *v*(*t*) is an additive ”noise” (the noise term can be the result of different parameters, such as thermal fluctuations).

The form of *K*_*i*_(*t*) in Eq. (1) depends on the choice of the *kernel* (or filter) for the membrane potential of the considered post-synaptic neuron. For instance, if we choose an exponentially decaying filter, then

2 $$ K_i(t) = \sum\limits_{t_f \in \mathcal{T}_i, t_f \leq t - d_i} e^{-\frac{t-t_f-d_i}{\tau_m}}, $$

where *τ*_*m*_ is the membrane time constant, *d*_*i*_ is the propagation delay between neuron *i* and the post-synaptic neuron and $\mathcal {T}_{i}$ is the set of firing times for the pre-synaptic neuron *i*.

The output, also called the *activity*, of the post synaptic neuron at time *t* will be

$$y(t) = f(h(t) - \theta), $$

where *f*(⋅) is the Heaviside step function and *𝜃* is the firing threshold. We also assume that after a firing, the membrane potential is *reset* back to the resting potential *h*_0_.

Another model of neurons that we also consider in this paper is the *stochastic* LIf model where the membrane potential is explained by Eq. (1) as before but the post-synaptic neuron’s activity is stochastic and is given by the following probability:

3 $$ \text{Pr}\{y(t) = 1\} = f_s(h(t) - \theta). $$

Here, *f*_*s*_(⋅) is *an increasing* function of its argument. There are several choices for *f*_*s*_(⋅) proposed in the literature in which the logistic function is perhaps one of the most popular. In this paper, however, we do not specify a particular function and only require that the function *f*_*s*_(⋅) is increasing in its argument.

### **Network model**

As for the network structure, we do not assume any specific topology on the neural graph. However, as is the case in many neural networks, we require a *balanced* network in terms of excitatory and inhibitory connections. This requirement ensures that the excitatory and inhibitory population act in such a way that the average activity stays below a threshold.

In that regard, we usually pick 80*%* of connections to be excitatory and 20*%* to be inhibitory. For numerical results, we set the weights of all excitatory connections to be + 1mV and that of an inhibitory connection to − *δ* mV, where *δ* = *n*_*exc*_/*n*_*inh*_, and *n*_*exc*_ and *n*_*inh*_ are the number of excitatory and inhibitory connections in the network. Also, in accordance with biological data and following Dale’s principle (Dale 1935), we fix the type of neurons to be either excitatory or inhibitory, which means all outgoing connections of a pre-synaptic neuron have the same sign. 2

We also assume that neural connections have intrinsic delays which represent the time it takes for the information to propagate through the axons and synapses. The delay for each link is assumed to be a random number in the interval (0,*d*_max_], where *d*_max_ > 0 is the maximum delay. The delays do not change, once assigned, they remain fixed.

### **Problem statement**

**Fig. 2:**
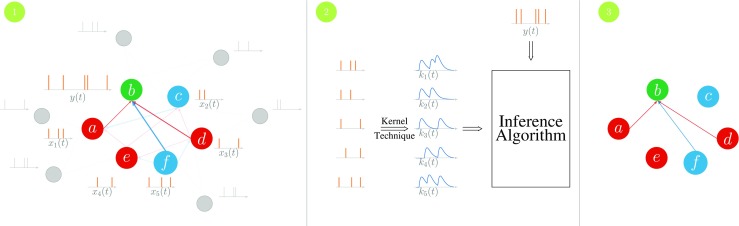
Network model: a recurrent neural network where we try to identify the incoming connections of node *b* by observing the spike trains $x_{1}(t), \dots , x_{5}(t)$ and *y*(*t*). Here we assume that edges have randomly chosen propagation delays and neurons can be excitatory or inhibitory. Note that there are some ”hidden” neurons (shown in gray) in the network as well and whose spiking activities are not recorded but they affect the membrane potential of the observed neurons. After applying some ”kernel” to account for the integration and leak in the membrane potential, we look for the set of weights that result in the best prediction of the output firing pattern, *y*(*t*). The result will ideally be as shown in Part 3. Note that in this paper we are not interested in reconstructing the exact weights (shown in Part 1 through the thickness of the lines), but to tell if two neurons are connected to each other and, if so, what the connection type is. If we repeat these steps for the incoming connections to other neurons, which can be done in parallel, we will get the complete connectivity graph

The goal of this paper is to propose a scalable learning algorithm that infers the (functional) *connectivity matrix* only by observing the firing activity of the neurons. Ideally, such an algorithm should be able to explain the observed firing activity as accurately as possible. Nevertheless, the ultimate goal of connectome mapping approaches is to identify the synaptic connectivity. As a result, we study the conditions under which the identified functional connectivity is a close approximation of the underlying synaptic connectivity. Specifically, we would like to design algorithms in order to identify the *type* of neural connections. In other words, an ideal inference algorithm should be able to find out if neuron *i* has a directed synaptic connection to neuron *j*, and if so, whether the connection is excitatory or inhibitory. Furthermore, this goal should be achieved by only observing the recorded firing activity. Figure 2 illustrates the model and the problem considered in this paper.

## The inference algorithms

We propose an iterative inference algorithm to identify the functional connections in a network of neurons based on their firing activity. To better explain the algorithm, we first study the simpler case of deterministic LIf neurons, as described in Eq. (1). We then show in a later section that how the proposed algorithm can be naturally extended to deal with stochastic neurons as well.

To start, let us assume that we are interested in identifying the incoming connections to one post-synaptic neuron (e.g. neuron *b* in Fig. 2). The following procedure can be then applied to every single neuron. In that case, note that we can re-write (1) in a vector form as

4 $$ u = Kg + v, $$

where $u \in \mathbb {R}^{T \times 1}$ is a vector whose *t*-th entry is *u*_*t*_ = *h*(*t*) − *h*_0_, $K \in \mathbb {R}^{T\times n}$ is a matrix whose (*t*,*i*)-th entry is *K*_*ti*_ = *K*_*i*_(*t*), $g\in \mathbb {R}^{n \times 1}$ is the vector of actual synaptic connection weights (i.e., *g* = [*g*_1_,…,*g*_*n*_]^⊤^) and $v\in \mathbb {R}^{T \times 1}$ is the noise vector. Without loss of generality, we assume *h*_0_ = 0 and *𝜃* = 0^3^. Now, let us define

5 $$ \hat{y}(t) = \left\{ \begin{array}{ll} + 1 & \text{if \(y(t) = 1\)};\\ -1 & \text{if \(y(t) = 0 \)};\end{array} \right. $$

where *y*(*t*) is the state of the post-synaptic neuron at time *t*. This way, we know that

$$K_t g + v(t) \geq 0,\ \forall t: \hat{y}(t) > 0 $$

and

$$K_t g + v(t) \leq 0,\ \forall t: \hat{y}(t) < 0, $$

where *K*_*t*_ is the *t*-th row of matrix *K*. By letting

$$\hat{Y}_{T \times T} = \text{diag}(\hat{y}(1),\dots,\hat{y}(T)), $$

we can rewrite the above constraints in a matrix form as follows

6 $$ \hat{Y}(Kg + v) > \textbf{0}, $$

where **0** is the all zero vector and the inequality is entrywise. Equation (6) is the cornerstone of our proposed algorithm. In order to find the neural connections, we aim to solve the following optimization problem

7 $$ \textit{\textbf{Problem I}:} \min_{w} \Vert w \Vert_{\ell} \quad\text{s.t. } \quad \hat{Y}K^{\prime} w > \textbf{0}. $$

Basically, by knowing the matrix $\hat {Y}$ and the firing activity of neurons, we will look for the smallest vector vector *w* (in *ℓ*-norm) that satisfies a set of constraints.

Also note that we used a different kernel matrix *K*^′^, which may or may not be the same as the true kernel matrix *K*, depending on our *prior knowledge* about the underlying neural model.

We will show that in scenarios where the original problem is feasible, i.e., when *Kg* > **0**, as long as *K*^′^ and *K* are close (in some precise algebraic sense) then by solving *Problem I*, given by Eq. (7), we will be able to find the type/sign and the location of non-zero entries in *g*, the vector of the underlying synaptic neural connections.

In practice, however, due to incoming traffic from hidden neurons as well as large membrane noise, *Problem I* may be infeasible, i.e., the constraints define an empty set. Therefore, to design a more practical algorithm we reformulate the problem as follows:

8 $$ \textit{\textbf{Problem II}:} \min_{w} \Vert w \Vert_{\ell} + \sum\limits_{t} L(A_t w), $$

where *A*_*t*_ is the *t*-th row of matrix *A*, defined as $A = \hat {Y}K^{\prime }$, and *L* is a convex loss function that penalizes unsatisfied constraints. This way, we look for a regularized solution *w*^∗^. Note that for a proper choices of *ℓ* (e.g. *ℓ* ≥ 1), the above problem is convex. The regularization helps the algorithm prevent overfitting as well as obtaining a more biologically-realistic set of weights, e.g. more sparse. However, the degree of regularization should be tuned as well since if we put more emphasize on regularization than the cost function and regularize the weights too much, the performance in predicting spikes will suffer.

There are many choices of loss functions used in the literature. One of the most well-known is hinge-loss, i.e.,

$$L(x) = \max(0,1-x). $$

For the rest of the paper, we will use the hinge loss as it is well-suited for our learning algorithm and there is a wealth of optimization techniques for efficiently solving the above regularized optimization problem.

### Centralized inference algorithms

To solve *Problem II*, given by Eq. (8), we propose two different *online* approaches, with emphasis on scalability and capability to deal with limited memory.

The first approach is an extension of the Perceptron-based algorithm we proposed in Karbasi et al. (2015) and focuses on solving the primal problem. In the proposed approach, we choose *ℓ* = 1 to favor sparsity in the connections. Then, by noting that the sub-gradient of the hinge loss function contains

$$L^{\prime}(x) = \left\{ \begin{array}{ll} -1 & \text{if } L(x) > 0;\\ 0 & \text{otherwise};\end{array} \right. $$

we derive the following update rule at iteration *τ* of the algorithm:

$$w(\tau+ 1) = w(\tau) + \gamma L(A_i w(\tau)) A_i^{\top}, $$

where *γ* is the learning rate.

Now, to take care of the sparsity regularization, let $\mathcal {F}(x,\eta )$ be the following soft-thresholding function

9 $$ \mathcal{F}(x,\eta) = \left\{ \begin{array}{ll} x-\eta & \text{if } x > \eta,\\ x+\eta & \text{if } x < - \eta,\\ 0 & \text{if } |x| < \eta .\end{array} \right. $$

Previous studies have shown that iteratively applying the soft-thresholding function above to our estimates *w*(*τ*) will result in a sparse solution (Wright et al. 2009; Goldstein et al. 2014). As a result, and by putting these steps together, the proposed approach, called NeuInf, is shown in Algorithm 1. 
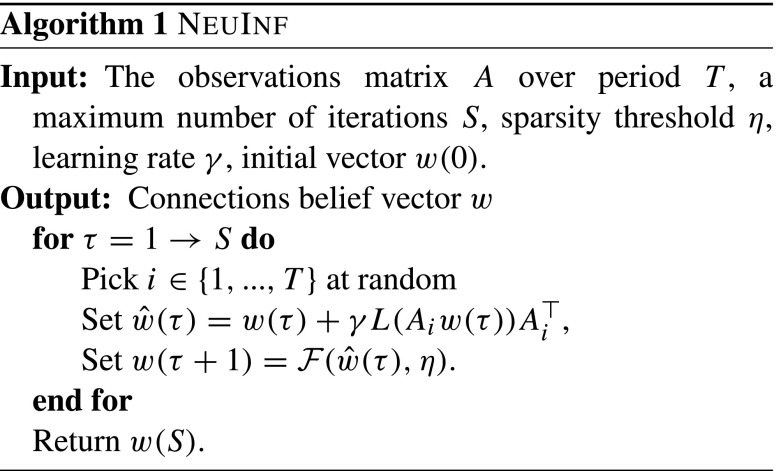


The second approach is based on solving the *dual* form of *Problem II* using the (Stochastic) Dual Coordinate Descent method. This particular formulation is specially interesting from the scalability viewpoint, as discussed in Jaggi et al. (2014). To this end, and to facilitate the formulation, we choose *ℓ* = 2 in this approach and use the soft-threshold function to trim the entries of the returned weight vector. To formulate the problem in its dual form, we use Fenchel’s conjugate of the loss function and the regularization term. The Fenchel dual of the *ℓ*_2_-norm is itself and that of the hinge loss is given by

$$L^{\star}(x) = \left\{ \begin{array}{ll} x & \text{if } -1<x < 0;\\ \infty & \text{otherwise}.\end{array} \right. $$

Therefore, we can formulate the dual problem as Jaggi et al. (2014):

10 $$\begin{array}{@{}rcl@{}} \max\limits_{\lambda \in [0,1]^T} E(\lambda) &=& - c \Vert A^{\top} \lambda \Vert_2^2 - \sum\limits_t L^{\star}(-\lambda_t) \\ &=& -c \Vert A^{\top} \lambda \Vert_2^2 + \sum\limits_t \lambda_t, \end{array} $$

where $\lambda \in \mathbb {R}^{T \times 1}$ is the vector of the dual variables and *c* is a positive constant to control the extent of regularization. By solving the above problem for the optimal dual variables, *λ*^∗^, we can then find the optimal set of weights as

11 $$ w^{\ast} = A^{\top} \lambda^{\ast}. $$

Thus, we can solve *Problem II* using the Stochastic Dual Coordinate Descent (SDCD) technique. The details are given in Algorithm 2 (Dual NeuInf). One strong point of the coordinate descent method is that it does not require to tune the learning rate as the decent step size, indicated by variable Δ*λ*, is selected automatically in the algorithm. This auto-tuning makes Dual NeuInf particularly attractive from the practical point of view. 
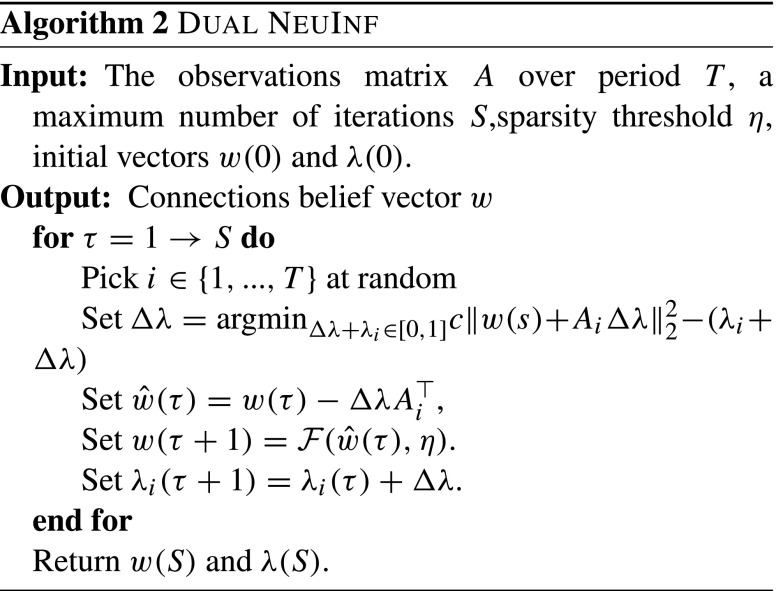


### Scalable inference algorithms

In practical situations, the matrix of observed neural activity could be very large, especially due to the large number of recorded samples, *T*. For instance, one of the datasets that we have used to evaluate the performance of the proposed algorithm has around *T* = 7,000,000 rows and *n* = 1000 columns, i.e., a matrix of size 7000000 × 1000. In some cases the dataset will be even larger. Fitting such large matrices into memory (RAM) is usually difficult due to the limited amount of available resources. Therefore, it is desirable to design an algorithm that can cope with the limits on memory. Furthermore, from the computational point of view, it would also be important to have an algorithm that can break the problem into smaller sub-problems, solve those sub-problems *in parallel*, and merge the results such that the overall solution is near-optimal.

**Fig. 3:**
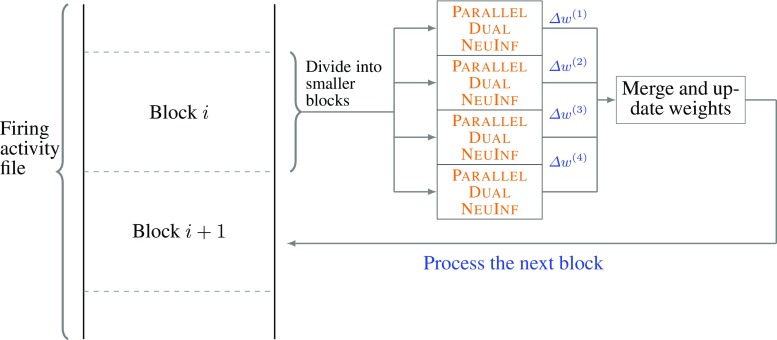
Parallelization architecture: our proposed algorithm reads smaller blocks of data from the file that contains the recorded activity, process them, updates the weights and move to the next block. Within each block, the task of processing and updating the weights is parallelized among multiple cores and the results are merged afterwards

To this end, we have also designed parallel versions of NeuInf and Dual NeuInf that can deal with limited memory and to fully utilize the available computational resources. We first divide the data matrix into *M* non-overlapping smaller blocks *A*^(1)^,…,*A*^(*M*)^, each of size (around) *T*/*M* × *n*. We can then apply either NeuInf or Dual NeuInf to solve the inference problem for each block and later merge the results. Depending on the amount of available resources, we either can do this process in parallel (i.e., if enough RAM is available) or do this process sequentially, loading one block *A*^(*i*)^ at a time into the memory (i.e., when RAM is limited). The proposed computational architecture is shown Fig. 3.

The detailed process for parallelizing NeuInf is given in Algorithm 3. For algorithms that are based on Dual Coordinate Descent methods, e.g., Algorithm 2, an elegant parallelization procedure is proposed by Jaggi et al. (2014), called Communication-Efficient Distributed Dual Coordinate Ascent (CoCoA). We will adapt this technique to parallelize Dual NeuInf. The details are shown in Algorithm 4. 
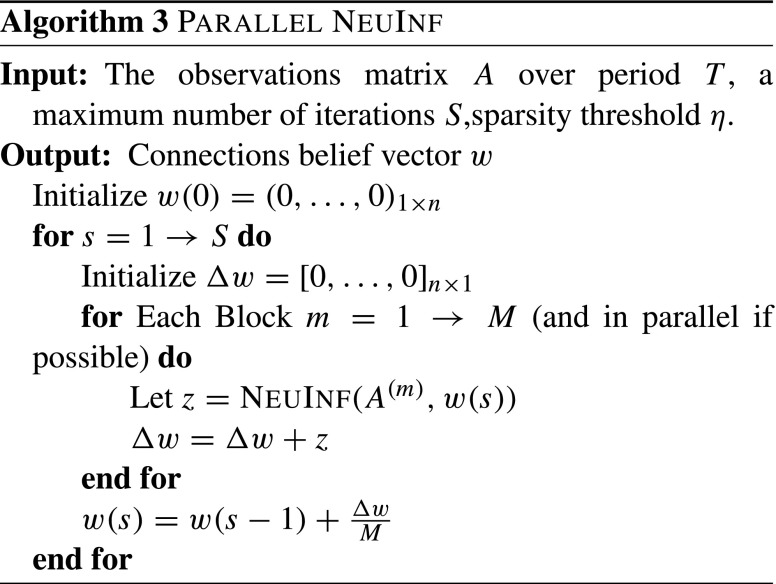
 
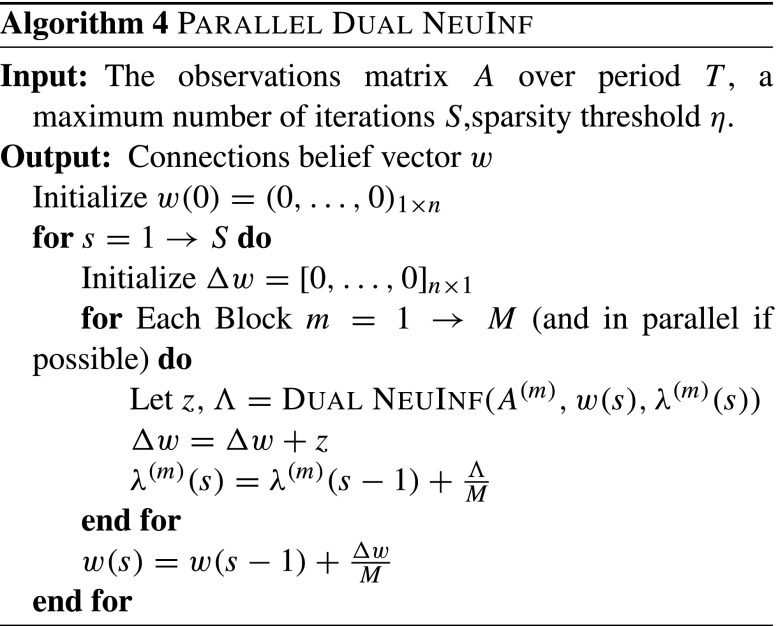


## Theoretical analysis

In this section, we analyze the performance of the proposed algorithms in order to identify connections under which the returned functional graphs closely approximates the underlying synaptic connections. We should again remark that our focus in this paper is to identify the existence and type of connections and not the corresponding weights.

We start by proving the desired results for a neural network consists of deterministic (and noisy) LIf neurons specified by Eq. (1). We also assume that there is incoming traffic from some unobserved (also called *hidden*) neurons. We establish sufficient conditions on both statistical properties of the noise as well as the *inference kernel* (denoted by *K*^′^) such that the type of connections in identified functional graph by the algorithms introduced in the previous section matches the type of corresponding neural connections in the underlying synaptic graph. We then extend our results to show that the same algorithm can be applied to the more realistic scenario of *stochastic* LIf neurons.

### Network of deterministic noisy LIf neurons with hidden traffic

For the network of deterministic noisy LIf neurons, we first show that as long as the noise term satisfy some statistical properties, the algorithm yields the desired result. We then investigate the conditions under which the net effect of incoming traffic from a set of hidden neurons can be modeled by the noise term with the specified statistical properties, which means that the algorithm will be successful in identifying the connection types even in presence of unobserved traffic.

To start, let us remind ourselves that the membrane potential of LIf neurons, define in Eq. (1), is given by

$$h(t) = h_0 + \sum\limits_{i = 1}^n g_i K_i(t) + v(t), $$

where *v*(*t*) is the added noise at time *t*. Intuitively, if the magnitude of noise is very small, then the set of constraints $\hat {Y}Kg>\textbf {0}$ will not be affected by noise. Likewise, if the noise has a zero mean, and we have enough firing data for the pre-synaptic neurons, we should be able to reconstruct the connections (by averaging out the noise) and in the limit of large data.

The following assumptions state the above intuition more rigorously. We then show that as long as these assumptions hold, we can identify the type of connections in the limit of large *T*.

1. **Having enough firing data**: the observed neurons fire at a rate linear in *T*, i.e., neuron *i* fires *α*_*i*_*T* spikes in the interval [0,*T*], with *α*_min_ > 0.
2. **Zero-mean noise**: the noise in the membrane potential {*v*(*t*)} is a zero-mean^4^ random variable and its samples are uncorrelated if they are more than Δ_*v*_ time slots apart.

Note that the first assumption makes sure that each neuron fires enough spikes for its connection to be successfully identified and the second assumption basically states that the noise should have a *vanishing correlation*, i.e., the very long future samples should not depend on the current samples. We also trivially pick the inference kernel *K*^′^ in such a way that it captures the quantity of the firing activity, i.e., the entries in the inference kernel matrix *K*^′^ are all non-negative and the number of non-zero entries in column *i* of the matrix *K*^′^ is proportional to the number of spikes fired by neuron *i*. For instance, we could use a kernel *K*^′^, based on the exponential decay function, where the entry $K^{\prime }_{ti}$ is equal to $e^{-(t-t_{f_{i}})}$, where $t_{f_{i}} < t$ is the last time neuron *i* had fired before time *t*. Lemma 1 provides a more general condition on the required statistical properties of matrix *K*^′^ and its relation to matrix *K* so that our proposed algorithm is capable of finding the type of connections under certain conditions.

#### **Lemma 1**

*Let us assume that we have enough samples such that the matrixK*^⊤^*Kand*(*K*^′^)^⊤^*K*^′^*are invertible. Now, if the matrixK*^⊤^*K*^′^*is positive definite, then, under the assumption A1-A2 defined above, and in the limit of large* T*, we can recoverthe type of the actual connections, i.e., the estimated weight vectorw*^∗^*(the output of Algorithm1 or 2) has the same sign as the actual connection weights, namely,*

$$\lim_{T \rightarrow \infty} \Pr\{ w^*_i g_i > 0 \} \rightarrow 1,\ \forall i = 1,\dots,n,\text{ where } |g_i| \neq 0. $$

#### (**The proof is given in Appendix**A.1)

The above theorem addresses the case of a deterministic noisy LIf neuron. Now we can use this result and extend it to a scenario where we have incoming traffic from a set of unobserved (hidden) neurons. To this end, suppose there are *m* unobserved neurons whose spikes affect the membrane potential of the post-synaptic neuron in consideration through a set of connections $g^{\prime } = [g^{\prime }_{1},\dots ,g^{\prime }_{m}]$. As a result, we can rewrite Eq. (4) as,

12 $$ u = Kg + Hg^{\prime}, $$

where $H \in \mathbb {R}^{T \times m}$ is the net effect of *m* outside neurons filtered through the neural kernel. Now, given the result of Lemma 1, we intuitively know that as long as *Hg*^′^ is a zero-mean random variable with vanishing correlation, we should be able to recover the type of connections. The following assumptions formulate this intuition more rigorously.

(A3) Traffic of two hidden/outside neurons *i* and *j* are independent of each other.
(A4) The incoming weights from the hidden/outside neurons form a *balanced random* network (similar to the incoming traffic from ”visible” neurons), i.e., $\mathbb {E}\{g_{i}^{\prime }\} = 0.$
(A5) Outgoing traffic of neuron *i* at time *t* and *t* + Δ are uncorrelated for sufficiently large Δ.

In words, assumptions A3 and A4 ensure that *Hg*^′^ is a zero-mean random variable with a vanishing correlation. Assumption A5 requires that the firing activity of each neuron has a *vanishing correlation*, i.e., very far ahead future spikes should be uncorrelated from current spikes. Out of the three, assumption A3 is probably the most strict one and assumption A5 is the weakest one as it is automatically satisfied when there is a *post-synaptic* spike in the interval [*t*,*t* + Δ) (due to the ”reset” effect of the membrane potential).

Note that a direct consequence of the above assumptions is that if we sample neurons at intervals that are far apart, the noise terms should be uncorrelated. This fact is useful in practice in order to design better algorithms.

Now, the following theorem shows that given the above assumptions, we can rewrite *Hg*^′^ as a zero-mean colored noise with a vanishing correlation.

#### **Lemma 2**

*Given assumptions A2-A5 above, the random variable* *v*(*t*) = *H*_*t*_*g*^′^ *form a colored random variable with a vanishing correlation.*

#### (**The proof is given in Appendix**A.2)

Combining the results of Lemmas 1 and 2, we can prove the convergence of the algorithm for the case of deterministic noisy LIf neurons with incoming hidden traffic. This is formally proven in the next theorem.

#### **Theorem 1**

*Let us assume that we have enough samples such that the matrix* *K*^⊤^*K* *and* (*K*^′^)^⊤^*K*^′^ *are invertible. Now, if the matrix* *K*^⊤^*K*^′^ *is positive definite, then, under the assumptions A1 through A5 stated above, and in the limit* *of large* *T, we can recover the type of the actual connections, i.e., the estimated weight vector* *w*^∗^ *has the same sign as the actual connection weights. formally,*

$$\lim_{T \rightarrow \infty} \Pr\{ w^*_i g_i > 0 \} \rightarrow 1,\ \forall i = 1,\dots,n,\text{ where } |g_i| \neq 0. $$

#### (**The proof is given in Appendix**A.3)

ᅟ

#### Network of stochastic LIf neurons

In the previous section, we proved that under certain assumptions our proposed algorithm are guaranteed to identify the type of connections in the limit of large data for deterministic LIf neurons (with hidden incoming traffic as well). In this section, we show that the same can be proven for stochastic LIf neurons if we slightly modify the proposed algorithm. The main idea is to show that solving the problem for stochastic neurons results in the same solution as solving the problem for deterministic neurons, defined in *Problem II*. Therefore, we can solve *Problem II* for the stochastic case as well to identify the connections.

To start, recall that the firing rule for stochastic LIf neurons, defined in Eq. (3), is given by

$$\text{Pr}\{y(t) = 1\} = f_s(h(t) - \theta), $$

where the membrane potential, *h*(*t*) is given by

$$h(t) = h_0 + \sum\limits_{i = 1}^n g_i K_i(t) + v(t). $$

From a statistical point of view, we can cast the neural network reconstruction as an instance of a Maximum Likelihood estimation: find a vector *w* that maximizes the likelihood of observing the output spike pattern {*y*(*t*)}, given the set of pre-synaptic spikes or their ”filtered” effect through the kernel matrix *K*^′^. More precisely, we are interested in solving the following problem:

13 $$ argmax_{w} \Pr\{ y|K^{\prime},w \}, $$

where $y\in \mathbb {R}^{T \times 1}$ is the vector of observed post-synaptic spikes and $K^{\prime } \in \mathbb {R}^{T \times n}$ is the neural kernel matrix that captures the leaky integrated effect of the pre-synaptic neurons. This is in fact what traditional GLM approaches do to identify the vector *w* (Paninski 2004). However, in this section, we show that under mild and natural assumptions on the post-synaptic neuron and its firing pattern, solving Problem (13) is equivalent to solving *Problem II*. By establishing this connection we can solve the above ML problem at scale, as we explained earlier.

The assumptions are as follows

1. The function *f*_*s*_(⋅) is an increasing function of its argument.
2. The firing pattern of the post-synaptic neuron has a *vanishing correlation*, i.e., if two samples are more than Δ time slots apart, they becoming conditionally independent. More precisely, if *t*^′^− *t* > Δ, then
  $$\begin{array}{@{}rcl@{}} \Pr\{y(t),y(t^{\prime})|K^{\prime},w\} &=& \Pr\{y(t)|K^{\prime}(0,t),w\}\\ &\times&\Pr\{y(t^{\prime})|K^{\prime}(t,t^{\prime}),w\}, \end{array} $$
  where *K*^′^(*t*_1_,*t*_2_) is the subset of samples in the interval [*t*_1_,*t*_2_].

Note that Assumption A2 will be easier to satisfy if the post-synaptic neuron has fired at least once in the interval [*t*,*t*^′^), due to the *reset* effect of neurons.

Under our assumptions, we know that if we only consider samples that are more than Δ time slots apart, they are independent. With slight abuse of notation, let $y \in \mathbb {R}^{T^{\prime } \times 1}$ and $K^{\prime } \in \mathbb {R}^{T^{\prime } \times n}$ denote the vector of sampled output spikes that are at least Δ samples apart and the corresponding kernel matrix, respectively. Then, one can rewrite the ML problem (13) as

14 $$ argmax_{w} \text{Pr}\{ y|K^{\prime},w \} = argmax_{w} \prod\limits_{t = 1}^{T^{\prime}} \text{Pr}\{ y(t)|K^{\prime}(t),w \} $$

or equivalently,

15 $$ argmax_{w} \sum\limits_{t = 1}^{T^{\prime}} \log\left( \text{Pr}\{ y(t)|K^{\prime}(t),w \}\right). $$

Let $T^{\prime }_{+}$ indicate the set of time instances such that $\forall t \in T^{\prime }_{+}$ we have *y*(*t*) = 1. Likewise, let $T^{\prime }_{0}$ be the set of instances such that *y*(*t*) = 0, for $\forall t \in T^{\prime }_{0}$. By combining Eqs. (1), (3), and (15) we obtain the following optimization problem to solve

16 $$ argmax_{w} \sum\limits_{t \in T^{\prime}_{+}} \log\left( f_s(K^{\prime}_{t}w)\right) + \sum\limits_{t \in T^{\prime}_{0}} \log\left( 1-f_s(K^{\prime}_{t} w)\right), $$

where $K^{\prime }_{t}$ is the *t*-th row of *K*^′^. To simplify the above equation, let $K^{\prime \prime }_{t}\in \mathbb {R}^{1 \times n}$ be a vector in such a way that the following holds:

$$1-f_s(K^{\prime}_{t} w) = f_s(K^{\prime\prime}_t w). $$

##### *Remark 1*

For the special case of *f*_*s*_ being the sigmoid function, we have $K^{\prime \prime }_{t} = -K^{\prime }_{t}$. This is the form that has been considered in the context of GLMs (Paninski 2004).

Now, let us define matrix $H \in \mathbb {R}^{T^{\prime } \times n}$ as follows

17 $$ H_t = \left\{ \begin{array}{ll} K^{\prime}_t & \text{if } t \in T^{\prime}_{1};\\ K^{\prime\prime}_t & \text{if } t \in T^{\prime}_{0};\end{array} \right. $$

where *H*_*t*_ is the *t*-th row of *H*. As a result, we can rewrite problem (16) as

18 $$ argmax_{w} \sum\limits_{t} \log\left( f_s(H_{t} w)\right) $$

Note that *w* is bounded in practice. This implies that the term ∥*Hw*∥_2_ is also bounded. Therefore we can formulate the above optimization problem as follows:

19 $$ argmax_{w: \Vert H w\Vert = 1 }\sum\limits_{t} \log\left( f_s(H_t w)\right). $$

Our main observation is that the optimization problem given by Eq. (19) and *Problem II* are equivalent, meaning that the maximizer of Eq. (19) is also the minimizer of *Problem II*, as long as we pick a loss function that is

- Decreasing, i.e., *L*(*x*) ≤ *L*(*y*) if *x* ≥ *y*.
- Satisfies the inequality log(*f*_*s*_(*x*)) ≤−*L*(*x*).

For instance, if *f*_*s*_(⋅) is the sigmoid function, then we can pick *L*(⋅) to be the sigmoid function or a slightly modified version of the hinge loss, e.g., *L*(*x*) = max(*𝜖*(1 − *x*),1 − *x*), where 0 < *𝜖* < 1 is a small.

##### **Theorem 2**

*Under assumptions B1-B2 above, and with a proper choice of the loss function, the problems given by* Eq. (19) *and* *Problem II are equivalent in the sense that the solution* *w*^∗^ *to* *Problem II is also the maximizer of* Eq. (19).

##### (**The proof is given in Appendix**A.4)

This equivalency has significant consequences. First, we can efficiently find the ML estimator for problem (13). Second, it also suggests that the convergence results for our deterministic algorithm (discussed earlier in this section) also apply to the stochastic family of neurons.

## Experiments

In this section we validate the performance of the proposed algorithm via numerical experiments on both artificially generated data as well as data recorded from real neurons. For the former, we have used the dataset generated by Zaytsev et al. (2015).^5^ Testing on artificially generated data has an advantage in having access to the underlying synaptic connectivity (ground truth) which allows benchmarking the performance of the proposed algorithm. We have also applied the inference algorithm to a dataset of real recordings from the multiple hippocampal areas in rats (Mizuseki et al. 2013; Mizuseki et al. 2009).^6^

### Results on simulated data

The dataset of artificially generated spikes contains the firing activity of 1000 *LIf neurons*, with a fixed firing threshold of 20mV and a random (and unknown) synaptic propagation delay of up to 2ms (Zaytsev et al. 2015). The network topology was recurrent and randomly generated.

We apply Parallel Dual NeuInf to the dataset and compare the returned weights for each neuron to the actual ones. We calculate the accuracy of the algorithm in terms of three measures:

1. Spike prediction accuracy: we verify the ability of the algorithm to predict output firing activity of the post-synaptic neuron (i.e., by solving *Problem I*), given the inferred connection weights and the firing activity of its neighbors (when *Problem I* is feasible).
2. Average *quality*: we take an average over all the returned weights for excitatory, inhibitory and void connections when solving *Problem II*. In the ideal case, these three averages should be well-separated and the returned weights should be concentrated around the means (i.e., variance should tend to zero).
3. Precision and recall: we then transform the analog association matrix returned by the algorithm to a ternary *adjacency* matrix of the graph. We measure how accurately the algorithm has identified excitatory, inhibitory and void connections by calculating *precision* and *recall* for each connection type.

**Fig. 4:**
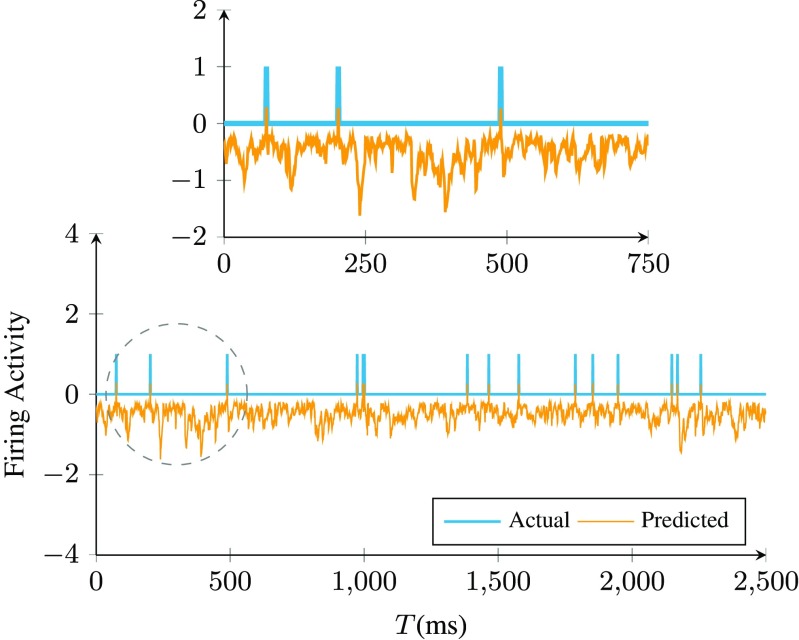
Sample of predicted vs, observed firing activity for neuron 1 in a recurrent network of 1000 LIf neurons

We start by evaluating the performance of Parallel Dual NeuInf in explaining observed firing activity. For relatively small *T*, the algorithm is always capable of finding a set of weights that accurately explain the observed activity (since *Problem I* remains feasible). Figure 4 illustrates a sample of observed vs. predicted spike activity by the set of weights returned by Parallel Dual NeuInf when the inference kernel *K*^′^ consists of a single decaying exponential filter with a time constant of 20ms.

**Fig. 5:**
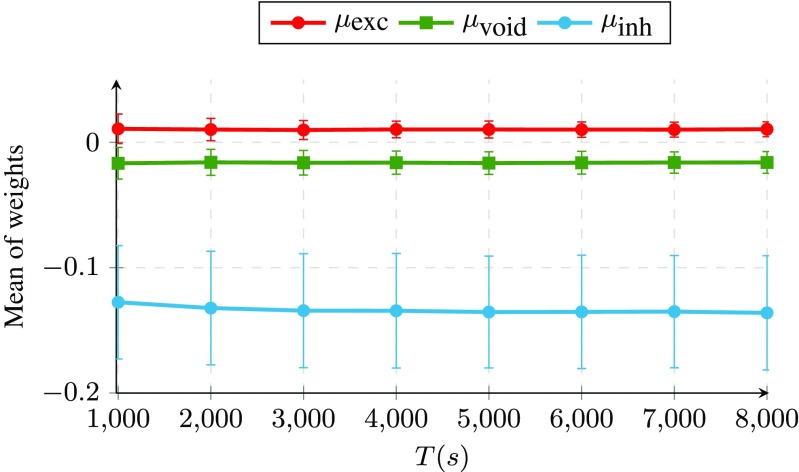
Average of weights of the incoming connections to neuron 1 returned by Parallel Dual NeuInf for each connection type as a function of the total number of recorded samples (*T*)

Moving to the quality of the returned weights in terms of matching with the underlying synaptic connections, we first calculated the average of returned weights for all excitatory, void and inhibitory connections, respectively. The desired properties that we are looking for are that, firstly, there should be an ordering between the average weights (excitatory to be higher than void, and void to be higher than inhibitory). Secondly, the variance of weights for each type should tend to zero as *T* grows, i.e., the algorithm returns a set of weights where the weights for each connection type are concentrated around their mean. Figure 5 shows that both of the desired properties hold for the proposed algorithm.

**Fig. 6:**
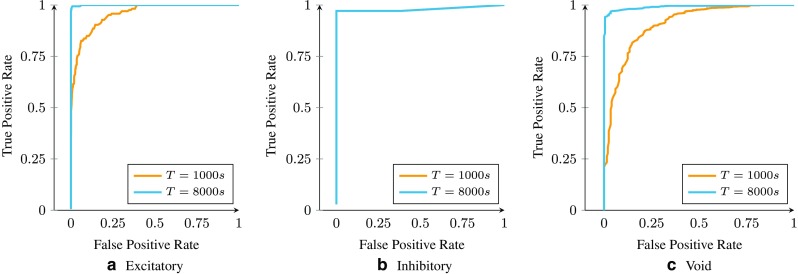
The ROC curve for the incoming connections to neuron 1 returned by Parallel Dual NeuInf

We also use the Receiver Operating Characteristic (ROC) curve to evaluate the quality of the returned weights. To this end, we normalize the incoming weights of neurons to zero mean and unit variance. We then gradually adjust two thresholds, one for excitatory and one for inhibitory connections, beyond which we declare a connection excitatory or inhibitory, respectively. For each pair of selected thresholds, the number of true and false positives for each connection type is calculated. The closer the area under the curve is to 1, the better the inference algorithm is. The results for the incoming connections to neuron 1 are shown in Fig. 6. As shown in the figure, having more samples result in more accurately inferred graphs. In Fig. 6, we also report the ROC curve for the ”aggregate inferred weights”, where we have performed the inference algorithm 5 times with different hyper parameters and averaged over the results. Clearly, this strategy results in a much better performance and an almost perfect reconstruction of connection types. We can make a trade off between the simulation time and RAM depending on the amount of available resources.

**Fig. 7:**
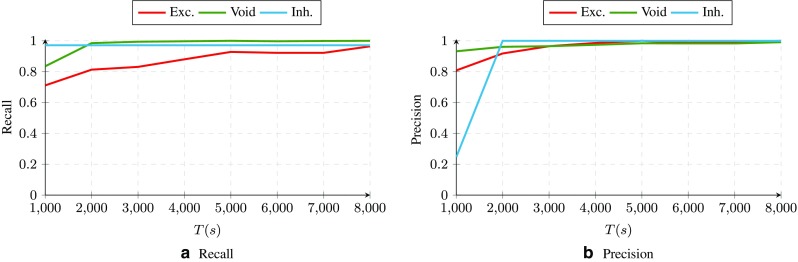
Precision and recall of for the incoming connections to neuron 1 in the artificially-generated dataset after transforming the returned association matrix by Parallel Dual NeuInf to a ternary adjacency matrix using the K-Means algorithm. We have averaged over several association matrices before transforming the results to ternary in order to reduce the effect of noise and randomness in the algorithm

Next, we evaluate the performance of the algorithm in terms of *precision* and *recall*. For this part, we use the aggregate set of weights discussed above and divide the set of incoming connections into three categories (i.e., excitatory, void and inhibitory), using the K-Means clustering algorithm (with 3 clusters). We then count the number of true positive/negatives for each connection type over this ternary adjacency matrix. This way, we can calculate the precision and recall of the algorithm as a function of *T* for the proposed algorithm, as shown in Fig. 7.

**Fig. 8:**
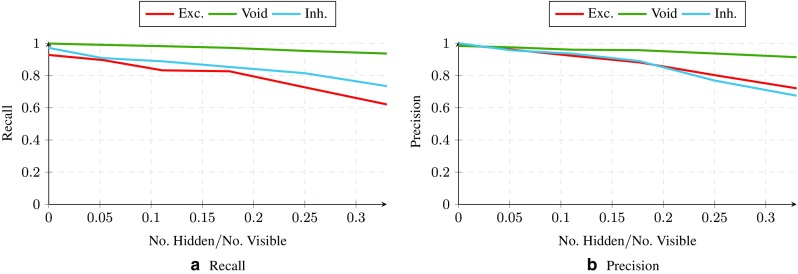
Effect of hidden neurons on the precision and recall of for the incoming connections to neuron 1 in the artificially-generated dataset. The horizontal axis shows the ration between the number of hidden and visible neurons in the network.The vertical axes show precision and recall, calculated after transforming the returned association matrix by Parallel Dual NeuInf to a ternary adjacency matrix using the K-Means algorithm

We have also examined the effect of hidden neurons on the performance of our proposed algorithm in identifying the connection types. Although hidden neurons were also present in the original dataset provided in Zaytsev et al. (2015), in order to quantitatively investigate the effect of hidden neurons, we artificially ”hid” some neurons in the database by removing their spike times when applying the proposed algorithm. We then evaluated the performance of the algorithm in correctly identifying the type of neural connections among the *visible* neurons. For a fixed number of hidden neurons, we generated 5 random graphs by randomly hiding the given number of neurons. The average results are shown in Fig. 8. In the figure, the horizontal axis illustrates the ratio of the number of hidden neurons to the number of visible neurons. The vertical axis show precision and recall for the excitatory, inhibitory and void connections. As shown in this figure, the algorithm is quite robust against the effect of hidden neurons and precision is less affected than recall. In other words, what the algorithm returns is quite accurate, but it might not capture all connections of the specific type when the number of hidden neurons is increasing.

**Fig. 9:**
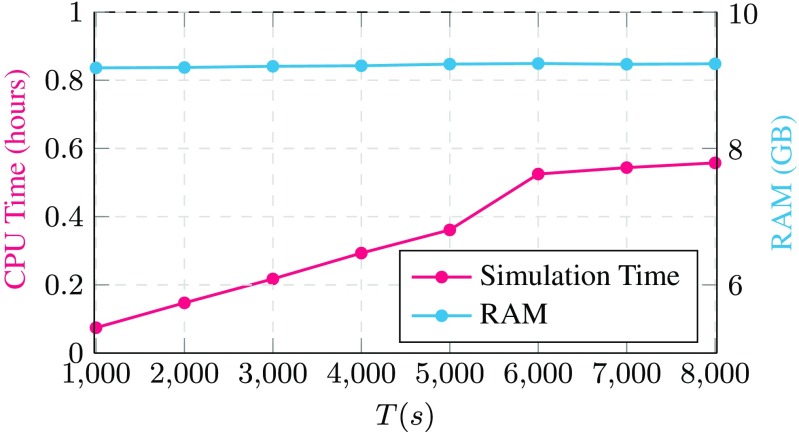
The amount of CPU and RAM used by the algorithm as a function of the number of recorded samples (*T*)

Finally, we calculated the amount of computational resources used by our algorithm. Figure 9 shows the simulation time (in hours) as well as the amount of RAM (in Gigabytes). As expected, since we divide the data matrix into smaller blocks and load them one at a time, the amount of RAM remains fixed and the simulation time scales (almost) linearly with the amount of available data.

### Results on real data

**Fig. 10:**
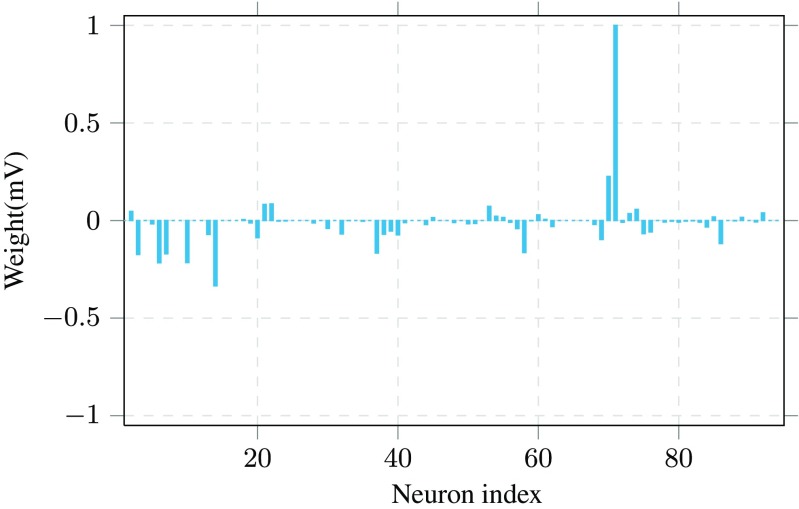
The incoming connections to neuron 1 in the dataset. The horizontal axis shows the index of the neuron that the connection is coming from and vertical axis indicates the magnitude of weight (in millivolts)

After validating the performance of the algorithm on simulated data, we applied the inference algorithm to recordings from the multiple hippocampal areas in rats (Mizuseki et al. 2013; Mizuseki et al. 2009). This dataset corresponds to 442 recording sessions when the rats were performing various tasks. In each session, the activity of tens of neurons were recorded simultaneously (ranging from 64 to 256 neurons). Here, and for most of available datasets of recordings from living species, the ground truth is not available. Therefore, we cannot benchmark the performance of the algorithm with respect to the underlying synaptic connectivity. Nevertheless, we can analyze the results in order to make sure that they are in line with biological findings about the species and perform sanity checks on the obtained functional graph. With this in mind, Fig. 10 illustrates the inferred weights by Parallel Dual NeuInf for neuron 1 based on the recordings for task ”ec013.18” in the dataset, that contains the firing activity of 94 neurons.

Furthermore, the data providers have also performed some physiological analysis to determine the type of each neuron (i.e., being excitatory or inhibitory). To better evaluate the performance of the proposed algorithm, we have also compared the “verdict” of our algorithm about the type neurons against the one found by the data providers. Note that there are several ways of deciding about the “type” of a neuron in our algorithm:

- One can calculate the “net” outgoing weight for each neuron and if it is higher/lower than a threshold, call it excitatory/inhibitory.
- Alternatively, one can count the number of positive and negative ”peaks” among the outgoing weights and classify the neuron as excitatory/inhibitory if these two numbers are significantly different from each other.

**Fig. 11:**
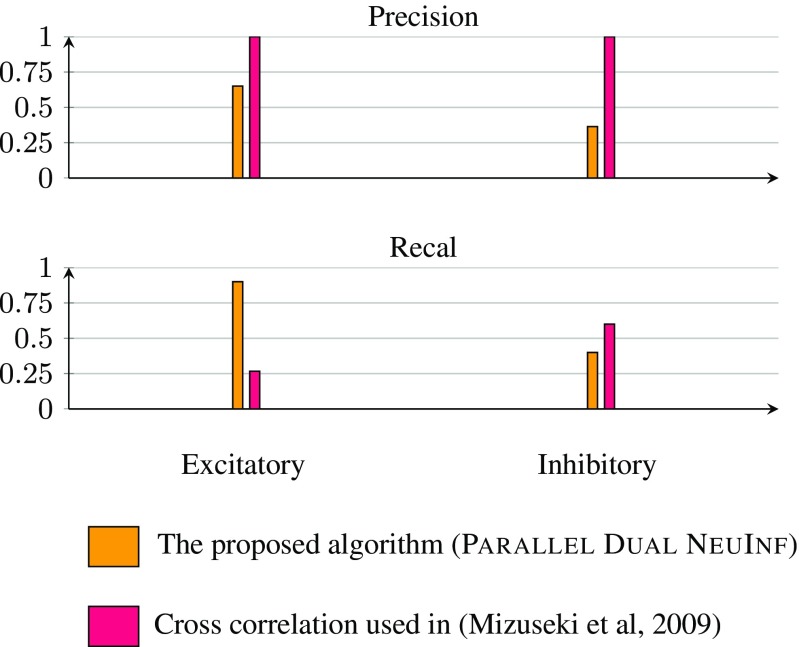
Comparison of neurons types determined by Parallel Dual NeuInf and cross correlation performed in Mizuseki et al. (2009). The results are benchmarked against the topological analysis made in Mizuseki et al. (2009) to decide the type of the neuron based on physiological evidence. As for the predictions made by Parallel Dual NeuInf, two different methods are considered for determining the type of a neuron based on its outgoing edges

We considered the second method and the results are shown in Fig. 11. To interpret the data given in Fig. 11, note that not all neurons were classified in Mizuseki et al. (2009) and we only compare the types for neurons that were indeed classified. In the figure, we have also included the results of neuron type prediction using cross correlation that was performed in Mizuseki et al. (2009). As shown in Fig. 11, the proposed algorithm performs quite well in identifying the excitatory neurons but requires improvements in identifying inhibitory neurons. This might be partly due to the fact that the firing rates of inhibitory neurons in the dataset was on average lower than those of excitatory neurons and the fact that the LIf model we considered in this paper is more accurate in modeling the behavior of excitatory neurons than that of inhibitory ones.

## Conclusion and future work

In this paper, we introduced a novel approach to identify neural connectivity from the observed firing activity of neurons. The proposed approach is based on a reformulation of LIf model for neurons in such a way that facilitates theoretical analysis and allows scalable implementations.

We theoretically proved the accuracy of our algorithm and derived the conditions under which the inferred functional connectivity matches that of the underlying synaptic network. We also showed that our algorithm is capable of dealing with both deterministic and stochastic LIf neurons through the same framework. Finally, using numerical analysis, we showed that the proposed algorithm successfully identifies the synaptic connections over a dataset of simulated spiking activity (to be able to benchmark against the ground truth) and is capable of dealing with datasets of real recordings yielding meaningful interpretations.

Different variations of the inference algorithm were proposed in this paper and each one is more suitable for certain scenarios in practice. In particular, for a centralized solution (when the data is not too big for a single machine), Dual NeuInf is a suitable algorithm as it does not require tuning a step size/learning rate. However, when the database is large, Parallel Dual NeuInf can be used to obtain the results more rapidly.

As for future directions, there are several major challenges that seems deeply intriguing. The first one concerns the existence of hidden neurons. In this paper we showed that as long as the incoming traffic from hidden neurons satisfy some statistical conditions, we are capable of finding the connectivity for the observed part of the network. Nevertheless, the more interesting challenge would be to (partially) identify the connectivity between the observed and hidden part of the network. The second challenge involves considering more realistic models of neurons. In this paper, we considered LIf neurons with fixed firing threshold. In reality though, the firing threshold is also adaptive and neurons need more accurate models to describe their behavior (especially the inhibitory ones). Taking these dynamical aspects is certainly part of our future work.

## Appendix A. Proofs of Theorems and Lemmas

Before we proceed to the proofs of the theorems in the paper, we prove a few auxiliary results that will help us obtain the desired proofs.

### **Lemma 3**

*Let* $r \in \mathbb {R}^{1\times T}$ *denote a non-zero vector and* *B and* *E be diagonal matrices with positive diagonal elements. Then, the matrix* *B* *r* ^⊤^ *r* *E* *is positive semidefinite.*

### *Proof*

To start the proof, let *C* = *r*^⊤^*r*. Now, note that $BC = B^{\prime } \bigodot C$,where $\bigodot $ indicates the Hadamard product and *B*^′^ = **1**_*T*×1_*b*_1×*T*_, with *b*_1×*T*_ being the diagonal entries of the matrix *B*. Also, let *E*^′^ = **1**_*T*×1_*e*_1×*T*_, with *e*_1×*T*_ being the diagonal entries of the matrix *E*.

Now, matrices *B*^′^and *E*^′^are positive semidefinite since their only non-zero eigenvalue is equal to the sumof all eigenvalues (the rest are zero), which is equal to the trace of matrix*B*^′^(resp. *E*^′^),i.e. ${\sum }_{j} b_{j} >0$(resp. ${\sum }_{j} e_{j} >0$).

Furthermore, the only non-zero eigenvalue of matrix C is equal to *r**r*^⊤^ > 0,which means matrix C is also positive semidefinite. Also, from Shcur product theorem (Schur 1911) weknow that the Hadamard product of two positive semidefinite matrices is positive semidefinite. Thus, matrix$D = B^{\prime } \bigodot C= BC$is positive semidefinite. Likewise, the matrix $D^{\prime } = D \bigodot E^{\prime }$is positive semidefinite, which proves the lemma. □

### **Lemma 4**

*Consider a banded matrix* *H in which only elements that are close the diagonal part are non-zero. More specifically,*

$$H_{ij} = \left\{\begin{array}{ll} 0, & \text{if } |i-j| > {\Delta},\\ \neq 0, & \text{if } |i-j| \leq {\Delta},\end{array} \right. $$

*for some constant integer* Δ > 0. *Furthermore, let* *h* = max_*i*,*j*_|*H*_*i**j*_|. *Then*,

$$\Vert H \Vert_2^2 \leq 8 {\Delta}^2 h^2 = \text{cons}. $$

### *Proof*

To start the proof, let *C* = *H*^⊤^*H*. Then,

20 $$ \Vert H \Vert_2^2 = \max\limits_{x} \Vert H x \Vert_2^2 = \max\limits_{x} x^{\top} H^{\top} H x = x^{\top} C x $$

where

21 $$ \Vert x \Vert_2 = 1 $$

Now for matrix *C* we have

$$C_{ij} = \left\{ \begin{array}{ll} 0, & \text{if } |i-j| > 2{\Delta},\\ \neq 0, & \text{if } |i-j| \leq 2{\Delta}.\end{array} \right. $$

As a result, and by letting *x*^(*d*)^be the shifted version of the vector *x* by *d* positions to the right and*c* = max *i**j**C*_*i**j*_, weobtain

22 $$\begin{array}{@{}rcl@{}} E(x) &=& \sum\limits_{i} \sum\limits_{j} x_{i} C_{ij} x_{j} \\ &=& \sum\limits_i x_{i} \sum\limits_{i-2{\Delta} \leq j \leq i + 2{\Delta}} C_{ij} x_{j} \\ &=& \sum\limits_i C_{ii} (x_i)^{2} + C_{i,i + 1} x_{i} x_{i + 1} + \dots\\ &&+ C_{i,i + 2{\Delta}} x_{i} x_{i + 2{\Delta}} \\ &&+ {\dots} + C_{i,i-1} x_{i} x_{i-1} + C_{i,i-2{\Delta}} x_{i} x_{i-2{\Delta}} \\ &\leq& c \sum\limits_{d = -2{\Delta}}^{2{\Delta}} x^{\top} x^{(d)} \\ &\leq& c \sum\limits_{d = -2{\Delta}}^{2{\Delta}} \Vert x \Vert_{2} \Vert x^{(d)} \Vert_{2} \\ &\leq& 4 {\Delta} c, \end{array} $$

where we have used the fact that ∥*x*∥_2_ = 1.Therefore,

23 $$ \Vert H \Vert_{2}^{2} \leq 4 {\Delta} c. $$

Now, letting *H*_*i*_ denote the *i*-th column of *H*, we obtain

$$c = \max\limits_{ij} C_{ij} = \max\limits_{ij} H_{i}^{\top} H_{j} \leq \Vert H_{i} \Vert_{2} \Vert H_{j} \Vert_{2}. $$

On theother hand, we have

$$\Vert H_j \Vert_2 \leq \sqrt{2 {\Delta} h^{2}}. $$

Therefore, *c* ≤ 2Δ*h*^2^. This implies that

$$\Vert H \Vert_{2}^{2} \leq 8 {\Delta}^{2} h^{2}, $$

which concludes the proof. □

### A.1 Proof of Lemma 1

The proof involves two main steps

1. Showing that mean value of $w^{\ast }_{i} g_{i}$ (over the noise’s probability distribution) is positive.
2. Showing the variance of $w^{\ast }_{i} g_{i}$ tends to zero as *T* grows.

To prove the first step, note that from solving *Problem II*, given by Eq. (8), we know that there is a vector *e* with positive entries where the optimal solution, *w*^∗^, satisfy (a subset of) the constraints, i.e.,

24 $$ \hat{Y}_cK_{c}^{\prime} w^{\ast} = e > \textbf{0}, $$

where $\hat {Y}_{c}$ and $K_{c}^{\prime }$ correspond to the set of satisfied constraints. As a result, and by letting $F^{\prime } = (K^{\prime }_{c})^{-1}$, we will have

$$w^{\ast} = F^{\prime} E\hat{y}_c, $$

where *E* is a diagonal matrix with positive entries, i.e., *E* = diag(*e*). Now from Eq. (6) we have

25 $$ Kg +v = B\hat{y}, $$

where *B* is a diagonal matrix with positive entries. By focusing only on the time instances where *w*^∗^ satisfy the constraints, we will see that

$$K_{c} g + v_{c} = B_{c}\hat{y}_{c}, $$

where *K*_*c*_ and *B*_*c*_ are the sub-matrices corresponding to the satisfied constraints. Therefore,

26 $$ g = F (B_{c}\hat{y}_{c}-v_{c}), $$

where $F = K_{c}^{-1}$. To simplify the notations, let *F*_*i*_ (resp. $F^{\prime }_{i}$) denote the *i*-th row of matrix *F* (resp. *F*^′^). Also, let $C_{T\times T} = F_{i}^{\top } F^{\prime }_{i}$. As a result, if *g*_*i*_ ≠ 0, we have

27 $$\begin{array}{@{}rcl@{}} g_i w^{\ast}_{i} &=& (\hat{y}_{c}^{\top} B_{c}-v_{c}^{\top}) F_{i}^{\top} F^{\prime}_{i} E \hat{y}_{c} \\ &=& \hat{y}_{c}^{\top} B_{c} F_{i}^{\top} F^{\prime}_i E\hat{y}_{c} -v_{c}^{\top} F_{i}^{\top} F^{\prime}_{i} E\hat{y}_{c}. \end{array} $$

Therefore,

28 $$\begin{array}{@{}rcl@{}} \mathbb{E}\{g_{i} w^{\ast}_{i}\} &=& \hat{y}_{c}^{\top} B_{c} F_{i}^{\top} F^{\prime}_{i} E\hat{y}_{c} -\mathbb{E}\{v_{c}^{\top}\} F_{i}^{\top} F^{\prime}_{i} E\hat{y}_{c} \\ &=& \hat{y}_{c}^{\top} B_{c} F_{i}^{\top} F^{\prime}_{i} E\hat{y}_{c}, \end{array} $$

where the last equality follows from the fact the we had a zero-mean noise. Now from Lemma 3 we know that the matrix $B F_{i}^{\top } F_{i} E$ is positive semidefinite. Therefore,

$$\hat{y}_{c}^{\top} B_{c} F_{i}^{\top} F^{\prime}_{i} E\hat{y}_{c}>0, $$

which means $\mathbb {E}\{g_{i}w^{\ast }_{i}\}>0$ if *g*_*i*_ ≠ 0.^7^

The second part of the proof relies on the fact that the variance of $g_{i} w^{\ast }_{i}$ tends to 0 as *T* →*∞*. To this end, let Δ_*v*_ denote the time window within which the noise term samples in the membrane potential remain correlated. Now, we have

29 $$\begin{array}{@{}rcl@{}} \sigma^{2}_{w_{i} g_{i}} &=& \mathbb{E}\{(w_{i} g_{i})^{2}\} - (\mathbb{E}\{w_i g_i\})^2 \\ &=& \left( \hat{y}_{c}^{\top} B_{c} F_{i}^{\top} F^{\prime}_{i} E\hat{y}_{c}\right)^{2} + \mathbb{E}\{ \left( v_{c}^{\top} F_{i}^{\top} F^{\prime}_{i} E \hat{y}_{c} \right)^{2}\} \\ &-& 2\mathbb{E}\{\left( \hat{y}_{c}^{\top} B_{c} F_{i}^{\top} F^{\prime}_{i} E \hat{y}_{c}\right)\left( v_{c}^{\top} F_{i}^{\top} F^{\prime}_{i} E \hat{y}_{c} \right)\} \\ &-& \left( \hat{y}_{c}^{\top} B_{c} F_{i}^{\top} F^{\prime}_{i} E\hat{y}_{c}\right)^{2} \\ &=&\hat{y}_{c}^{\top} E (F^{\prime}_{i})^{\top} F_{i} \mathbb{E}\{v_{c} v_{c}^{\top} \} F_{i}^{\top} F^{\prime}_{i} E \hat{y}_{c}, \end{array} $$

where we have used the fact that {*v*(*t*)} is a zero-mean random variable. Now let $H = \mathbb {E}\{v_{c} v_{c}^{\top } \} $. Then, and using the results of Lemma 4, we will get

30 $$\begin{array}{@{}rcl@{}} \sigma^{2}_{g_{i} w^{\ast}_{i}} &=& \hat{y}_{c}^{\top} E (F^{\prime}_{i})^{\top} F_{i} H F_{i}^{\top} F^{\prime}_{i} E \hat{y}_{c} \\ &\leq& \Vert H \Vert_{2} \Vert F_{i}^{\top} F^{\prime}_{i} E \hat{y}_{c} {\Vert_{2}^{2}} \\ &=& \sqrt{8} \sigma^{2}_{\max} {\Delta}_{v} \Vert F_{i}^{\top} F^{\prime}_{i} E \hat{y}_{c} {\Vert_{2}^{2}} \\ &\leq& \sqrt{8} \sigma^{2}_{\max} {\Delta}_{v} \Vert F_{i}^{\top} F^{\prime}_{i} {\Vert_{2}^{2}} \Vert \hat{y}_{c} {\Vert_{2}^{2}} \Vert E {\Vert_{2}^{2}} \\ &=& \sqrt{8} \sigma^{2}_{\max} {\Delta}_{v} e^{2}_{\max} T_{c}\Vert F_{i}^{\top} F^{\prime}_{i} {\Vert_{2}^{2}}, \end{array} $$

where *T*_*c*_ is the number of satisfied constraints (i.e. the length of $\hat {y}_{c}$), $\sigma ^{2}_{\max }$ is the maximum value in the matrix *H* and *e*_max_ is the maximum entry in the diagonal matrix *E*. At this point, note that

31 $$\begin{array}{@{}rcl@{}} \Vert F_{i}^{\top} F^{\prime}_{i} {\Vert_{2}^{2}} &\leq& \Vert F_{i} {\Vert_{2}^{2}} \Vert F^{\prime}_{i} {\Vert_{2}^{2}} \\ &\leq& \Vert F {\Vert_{2}^{2}} \Vert F^{\prime} {\Vert_{2}^{2}} \\ &=& \left( \lambda_{\max} (F^{\top} F) \right) \left( \lambda_{\max} ((F^{\prime})^{\top} F^{\prime}) \right), \end{array} $$

where *λ*_max_(*F*^⊤^*F*) is the maximum eigenvalue of the matrix *F*^⊤^*F*. Now, since *F* is the pseudo-inverse of the kernel matrix *K*_*c*_, we have $F = (K_{c}^{\top } K_{c})^{-1} K_{c}^{\top }$. Therefore,

$$\begin{array}{@{}rcl@{}} \lambda_{\max} (F^{\top} F) &=& \lambda_{\max} (F F^{\top}) \\ &=& \lambda_{\max} ((K_{c}^{\top} K_{c})^{-1} K_{c}^{\top} K_{c} ((K_{c}^{\top} K_{c})^{-1})^{\top})\\ &=& \lambda_{\max} ((K_{c}^{\top} K_{c})^{-1})\\ &=& \frac{1}{\lambda_{\max}(K_{c}^{\top} K_{c})}. \end{array} $$

Likewise, we can show that

$$\lambda_{\max} ((F^{\prime})^{\top} F^{\prime}) = \frac{1}{\lambda_{\max}((K_{c}^{\prime})^{\top} K_{c}^{\prime})}. $$

As a result, will obtain

32 $$ \sigma^{2}_{g_{i} w^{\ast}_{i}} \leq \frac{\sqrt{8}\sigma^{2}_{\max} e^{2}_{\max} {\Delta}_{v} T_{c}}{\lambda_{\max}(K_{c}^{\top} K_{c})\lambda_{\max}((K_{c}^{\prime})^{\top} K_{c}^{\prime})}. $$

We know that for any real-valued symmetric matrix *M*_*n*×*n*_, we have

$$\forall \nu \in \mathbb{R}^{n}, \Vert \nu \Vert_{2} = 1: \nu^{\top} M \nu \leq \lambda_{\max}. $$

We set $\nu = \frac {1}{\sqrt {n}}\textbf {1}_{n \times n}$, and by using $M = K_{c}^{\top } K_{c}$, we get

$$\lambda_{\max} \geq \frac{1}{n} \sum\limits_{ij} M_{ij} \geq \frac{1}{n} \text{Tr} (M), $$

where the last inequality follows from the fact that entries of matrix *M* (and those of *K*) are all non-negative. Now we have

33 $$\begin{array}{@{}rcl@{}} \text{Tr}(M) &=& \sum\limits_{i = 1}^{n} \sum\limits_{j = 1}^{T_c} ((K_{c})_{ji})^{2} \\ &\geq& \sum\limits_{i = 1}^{n} \alpha_{i} T_{c} \\ &\geq& \alpha_{\min} n T_{c}, \end{array} $$

where the first inequality follows from the fact that the term ${\sum }_{j = 1}^{T_{c}} ((K_{c})_{ji})^{2}$ is at least as large as the number of spikes fired by neuron *i* in the interval [0,*T*_*c*_]. Combining the above equations, we obtain

$$\lambda_{\max}(K_{c}^{\top} K_{c}) \geq \alpha_{\min} T_{c}. $$

Similarly, we can also show that

$$\lambda_{\max}((K_{c}^{\prime})^{\top} K_{c}^{\prime}) \geq \alpha_{\min} T_{c}. $$

Combined with Eqs. (29), (30) and (31), this results in

34 $$\begin{array}{@{}rcl@{}} \sigma^2_{w_i g_i} &\leq& \frac{\sqrt{8} \sigma^2_{\max} e^2_{\max} {\Delta}_v T_c}{\left( \alpha_{\min}T_c \right)^2} \\ &\leq& \left( \frac{2\sigma_{\max} e_{\max}}{\alpha_{\min}}\right)^2 \frac{{\Delta}_v }{T_c}. \end{array} $$

This shows the desired result that the variance tends to zero as *T*_*c*_ grows. Given that the number of satisfied constraints also grow with *T*, the above steps show that the variance tends to zero as *T* grows.

At this point, and with the above results, we can easily prove the theorem using the Chebyshev’s inequality. We simply define $\mu = \mathbb {E}\{ g_{i} w^{\ast }_{i}\}$ and will have

$$\text{Pr}\{ \vert g_{i} w^{\ast}_{i}- \mu\vert \geq k\sigma \} \leq \frac{1}{k^{2}}, $$

where $\sigma = \mathbb {E}\{ (g_{i} w^{\ast }_{i})^{2} \}$ and *k* is a constant bigger than one. Now since we know that $\lim _{T\rightarrow \infty } \sigma = 0$, we can select the constant *k* arbitrarily large, which is equivalent to having

35 $$ \lim_{T\rightarrow \infty} \text{Pr}\{ \vert w^{\ast}_{i} g_{i} - \mu\vert 0 \} \leq \epsilon, $$

for some arbitrarily small *𝜖* > 0. This shows that if *g*_*i*_ ≠ 0, the term *w*_*i*_*g*_*i*_ will be concentrated around its mean, *μ* > 0, with probability 1. This proves the theorem.

#### *Remark 2*

Note that the above theorem only states that if there is an underlying synaptic connection, i.e. if *g*_*i*_ ≠ 0, we will identify its sign in the limit of large data. However, it does not discuss the cases where *g*_*i*_ = 0. In those cases, the additional sparsity regularizes (e.g. in the form of *ℓ*_1_-normminimization) help us prune the connections by removing those that are close to zero.

### A.2 Proof of Lemma 2

We start by calculating $\mathbb {E}\{ v(t)v(t-{\Delta })\}$ for some integer Δ > 0, noting that $v(t) = {\sum }_{i = 1}^{m} H_{ti} g^{\prime }_{i}$.

$$\begin{array}{@{}rcl@{}} \mathbb{E}\{ v(t)v(t-{\Delta})\} &=& \sum\limits_{i} \sum\limits_{j} \mathbb{E}\{ H_{ti} H_{t-{\Delta},j} g^{\prime}_i g^{\prime}_j \}\\ &=& \sum\limits_{i} \mathbb{E}\{ H_{ti} H_{t-{\Delta},i} (g^{\prime}_i)^2 \}\\ &+& \sum\limits_{i} \sum\limits_{j \neq i} \mathbb{E}\{ H_{ti} H_{t-{\Delta},j} g^{\prime}_i g^{\prime}_j \}\\ &\stackrel{a}{=}& \sum\limits_{i} \mathbb{E}\{ H_{ti} H_{t-{\Delta},i} (g^{\prime}_i)^2 \}\\ &+& \sum\limits_{i} \mathbb{E}\{ H_{ti} g^{\prime}_i \} \sum\limits_{j \neq i} \mathbb{E}\{ H_{t-{\Delta},j} g^{\prime}_j \}, \end{array} $$

where $\overset {a}{=}$ follows from assumption A3. Hence,

$$\begin{array}{@{}rcl@{}} \mathbb{E}\{ v(t)v(t-{\Delta})\} &=& \sum\limits_{i} \mathbb{E}\{ H_{ti} H_{t-{\Delta},i} (g^{\prime}_i)^2 \} \\ &+&\sum\limits_{i} \mathbb{E}\{ H_{ti} g^{\prime}_i \} c_i \\ &\stackrel{b}{=}& \sum\limits_{i} \mathbb{E}\{ H_{ti} H_{t-{\Delta},i} (g^{\prime}_i)^2 \} \\ &-& \sum\limits_{i} \mathbb{E}\{ H_{ti} g^{\prime}_i\} \mathbb{E}\{ H_{t-{\Delta},i} g^{\prime}_i\}, \end{array} $$

where $c_{i} = \left ({\sum }_{j} \mathbb {E}\{H_{t-{\Delta },j} g^{\prime }_{j} \} - \mathbb {E}\{ H_{t-{\Delta },i} g^{\prime }_{i}\} \right )$ and $\stackrel {b}{=}$ follows from assumptions A3 and A2 since ${\sum }_{j} \mathbb {E}\{ H_{t-{\Delta },j} g^{\prime }_{j} \} = \mathbb {E}\{v(t-{\Delta })\} = 0$. At this point, let $\mathcal {E}_{t,{\Delta }}$ denote the event of having at least one post-synaptic firing in the interval [*t* −Δ,*t*] and $\bar {\mathcal {E}}_{t,{\Delta }}$ denote the event of having no post-synaptic firing in the interval [*t* −Δ,*t*]. Now, if we assume an exponentially decaying filter for membrane potential with a decay coefficient of *τ*, we obtain^8^

36 $$ H_{ti} = \left\{ \begin{array}{ll} e^{-{\Delta}/\tau} H_{t-{\Delta},i} + e^{d_i/\tau} {\sum}_{t-{\Delta} < t_{i} < t} e^{-\frac{t-t_i}{\tau}},& \text{if } \bar{\mathcal{E}}_{t,{\Delta}},\\ e^{d_{i}/\tau} {\sum}_{t_{f} < t_{i} < t} e^{-\frac{t-t_{i}}{\tau}}, & \text{if } \mathcal{E}_{t,{\Delta}}. \end{array} \right. $$

Therefore, and by letting

$$p_{\text{fire}} = \text{Pr}\{\text{a post-synaptic firing in } \in [t-{\Delta},t]\}, $$

from Eq. (36) we obtain

37 $$\begin{array}{@{}rcl@{}} \mathbb{E}\{H_{ti} \} &=& (1-p_{\text{fire}}) e^{-{\Delta}/\tau} \mathbb{E}\{H_{t-{\Delta},i} \} \\ &+& (1-p_{\text{fire}})\mathbb{E}\{S_1\} + p_{\text{fire}} \mathbb{E}\{S_2\}, \end{array} $$

where

$$\begin{array}{@{}rcl@{}} S_{1} &=& e^{d_{i}/\tau} \sum\limits_{t-{\Delta} < t_{i} < t} e^{-\frac{t-t_i}{\tau}},\\ S_{2} &=& e^{d_{i}/\tau} \sum\limits_{t_{f} < t_{i} < t} e^{-\frac{t-t_{i}}{\tau}}. \end{array} $$

Furthermore, we have

38 $$\begin{array}{@{}rcl@{}} \mathbb{E}\{H_{ti} H_{t-{\Delta},i} \} &=& (1-p_{\text{fire}}) e^{-{\Delta}/\tau} \mathbb{E}\{(H_{t-{\Delta},i})^2 \} \\ &+& (1-p_{\text{fire}})\mathbb{E}\{S_1\} \mathbb{E}\{H_{t-{\Delta},i} \} \\ &+& p_{\text{fire}} \mathbb{E}\{S_2\} \mathbb{E}\{H_{t-{\Delta},i} \}, \end{array} $$

where we have used assumption A5. As a result, and combining Eqs. (36), (37) and (38) we obtain

39 $$\begin{array}{@{}rcl@{}} \mathbb{E}\{ v(t)v(t-{\Delta})\} &=& \sum\limits_{i = 1}^{m} \mathbb{E}\{(H_{t-{\Delta},i})^{2}\}c^{\prime}_{i} \\ &-& \sum\limits_{i = 1}^{m} (\mathbb{E}\{H_{ti} H_{t-{\Delta},i} \})^{2} c^{\prime}_{i} \\ &=& e^{-{\Delta}/\tau} (1-p_{\text{fire}}) \sum\limits_{i = 1}^{m} \sigma^2_{i} \mathbb{E}\{(g^{\prime}_i)^{2}\}, \end{array} $$

where

$$c^{\prime}_{i} = \mathbb{E}\{(g^{\prime}_{i})^{2}\} e^{-{\Delta}/\tau} (1-p_{\text{fire}}), $$

and

$$\sigma^{2}_{i} = \mathbb{E}\{(H_{t-{\Delta},i})^{2}\}-(\mathbb{E}\{H_{ti} H_{t-{\Delta},i} \})^{2}. $$

In the above equation, both *e*^−Δ/*τ*^ and 1 − *p*_fire_ are decreasing functions of Δ (i.e., the time interval). Therefore, the correlation is vanishing which proves the lemma.

### A.3 Proof of Theorem 1

The proof is actually a direct consequence of Lemmas 1 and 2: Lemma 2 makes sure that, given assumptions A3-A5, the contribution of outside traffic satisfies the assumptions about the noise term in Lemma 1. Combined with assumptions A1-A2, we can then apply the proof of Lemma 1 to prove the desired result here.

### A.4 Proof of Theorem 2

To start the proof, let *e* = *H*_*t*_*w*. Then, we note that we can rewrite Problem II and the problem defined by Eq. (19) as

40 $$ \max\limits_{e, \Vert e \Vert_{2} = 1} E_{1} = \sum\limits_t \log\left( f_{s}(e)\right) $$

and

41 $$ \max\limits_{e, \Vert e \Vert_{2} = 1} E_{2} = - \sum\limits_{t} L(e), $$

where *e* = [*e*_1_,…,*e*_*T*_]^⊤^ and *L*(⋅) is a “suitable” cost function, i.e., it is decreasing and log(*f*_*s*_(*x*)) ≤−*L*(*x*).

Our goal is to show that the above problems have the same maximizer. The proof consists of two steps

1. Showing that both functions *E*_1_ and *E*_2_ are increasing functions of *e*.
2. Showing that *E*_1_ ≤ *E*_2_.

The first part follows from the fact that

$$\frac{\partial E_{1}}{\partial e} = \frac{f_{s}^{\prime}(e)}{f_{s}(e)} \geq 0, $$

where the inequality follows from the fact that 0 ≤ *f*_*s*_(*x*) ≤ 1 and *f*_*s*_(*x*) is an increasing function of *x*, therefore its derivative is always positive. Additionally, since *L*(*x*) is a decreasing function, *E*_2_ is also increasing.

Furthermore, note that since we have selected *L*(*x*) to be less than − log(*f*_*s*_(*x*)), then we will have

$$E_{1} \leq E_{2}. $$

All this results in the fact that the maximizer of function *E*_2_ will also maximize *E*_1_.

Now, given that the objective functions for both Problem (40) and Problem (41) are increasing, the maximizer of both are at the boundaries. Therefore, in order to solve the ML problem given in Eq. (13), we can focus on solving the potentially simpler problem, namely *Problem II* specified by Eq. (8). This concludes the proof.

## Footnotes

1 We sincerely thank Dr. Yury Zaytsev, Prof. Abigail Morrison and Dr. Moritz Deger for making their data and code publicly available.

2 Note that Dale’s law was only applied in generating firing data during numerical analysis and was not considered in designing the inference algorithm to identify the functional graph as it makes parallelization of the algorithm very difficult. Also previous work suggest that the effect of Dale’s law on the performance of the inference algorithms is usually marginals

3 Both these terms can be easily integrated into the weight vector by appending a separate entry to the vector *g* and the kernel matrix *K*. We focus on the case where *𝜃* = *h*_0_ = 0 for simplicity.

4 Even if the noise has a non-zero mean, it might be possible to compensate for that by adjusting the firing threshold in such a way that the mean of noise remain zero.

5 We sincerely thank Dr. Yury Zaytsev, Prof. Abigail Morrison and Dr. Moritz Deger for making their data and code publicly available.

6 We sincerely thank Prof. Kenji Mizuseki, Prof. Anton Sirota, Prof. Eva Pastalkova and Prof. György Buzsáki for making the dataset publicly available.
